# The Hsc/Hsp70 Co-Chaperone Network Controls Antigen Aggregation and Presentation during Maturation of Professional Antigen Presenting Cells

**DOI:** 10.1371/journal.pone.0016398

**Published:** 2011-01-20

**Authors:** Nadja Kettern, Christian Rogon, Andreas Limmer, Hansjörg Schild, Jörg Höhfeld

**Affiliations:** 1 Institute for Cell Biology, Rheinische Friedrich-Wilhelms-University Bonn, Bonn, Germany; 2 Institute for Molecular Medicine and Experimental Immunology, University Hospital Bonn, Bonn, Germany; 3 Institute for Immunology, University of Mainz, Mainz, Germany; University Paris Sud, France

## Abstract

The maturation of mouse macrophages and dendritic cells involves the transient deposition of ubiquitylated proteins in the form of dendritic cell aggresome-like induced structures (DALIS). Transient DALIS formation was used here as a paradigm to study how mammalian cells influence the formation and disassembly of protein aggregates through alterations of their proteostasis machinery. Co-chaperones that modulate the interplay of Hsc70 and Hsp70 with the ubiquitin-proteasome system (UPS) and the autophagosome-lysosome pathway emerged as key regulators of this process. The chaperone-associated ubiquitin ligase CHIP and the ubiquitin-domain protein BAG-1 are essential for DALIS formation in mouse macrophages and bone-marrow derived dendritic cells (BMDCs). CHIP also cooperates with BAG-3 and the autophagic ubiquitin adaptor p62 in the clearance of DALIS through chaperone-assisted selective autophagy (CASA). On the other hand, the co-chaperone HspBP1 inhibits the activity of CHIP and thereby attenuates antigen sequestration. Through a modulation of DALIS formation CHIP, BAG-1 and HspBP1 alter MHC class I mediated antigen presentation in mouse BMDCs. Our data show that the Hsc/Hsp70 co-chaperone network controls transient protein aggregation during maturation of professional antigen presenting cells and in this way regulates the immune response. Similar mechanisms may modulate the formation of aggresomes and aggresome-like induced structures (ALIS) in other mammalian cell types.

## Introduction

**Figure 1:**
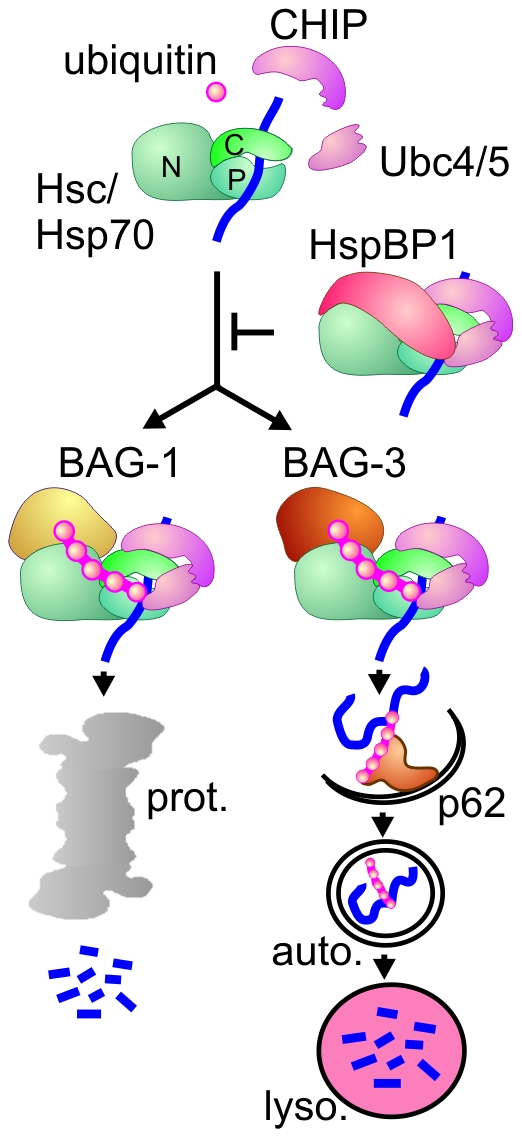
The co-chaperones CHIP, BAG-1, BAG-3, and HspBP1 regulate chaperone-assisted degradation. Chaperone-assisted degradation is initiated when CHIP binds to the carboxy-terminus of Hsc/Hsp70 (‘C’). After CHIP-mediated recruitment of ubiquitin conjugating enzymes of the Ubc4/5 family, the chaperone-bound protein substrate is modified by attachment of a ubiquitin chain. The co-chaperones BAG-1, BAG-3 and HspBP1 regulate CHIP function. They are able to bind to the amino terminal ATPase domain of Hsc/Hsp70 (‘N’) at the same time when CHIP occupies the carboxy-terminus. Binding of BAG-1 initiates sorting to the proteasome (‘prot.’), whereas BAG-3 triggers the recruitment of the autophagic ubiquitin adaptor p62 and thus facilitates substrate degradation through the autophagosome-lysosome pathway. In contrast, HspBP1 inhibits the ubiquitin ligase activity of CHIP in the formed chaperone complex and thereby abrogates chaperone-assisted degradation. (‘P’ - peptide binding domain of Hsc/Hsp70; ‘auto.’ - autophagosome; ‘lyso.’ - lysosome).

Living cells employ a sophisticated machinery for maintaining their proteome. This protein homeostasis (proteostasis) machinery balances protein synthesis, folding and degradation in a manner adjustable to alterations in the inherited proteome, to physiological stimuli and to environmental insults [1]. Impaired proteostasis can lead to protein aggregation that is detrimental or toxic to cells, causing for example severe neurodegenerative diseases such as Parkinson's disease [2]. Molecular chaperones of the Hsp70 family are key components of the cellular proteostasis machinery because they fulfill a dual function during protein quality control. They facilitate protein folding and assembly whenever possible, but are also able to direct folding incompetent clients towards degradation [3], [4], [5]. Constitutively expressed Hsc70 and stress inducible Hsp70 represent the main family members in the mammalian cytoplasm and nucleus. Their activity is regulated by a network of co-chaperones that modulate the ATP-dependent peptide binding cycle of the chaperones and/or facilitate a cooperation with other protein complexes, chaperones, or degradation systems [6]. With regard to chaperone-assisted degradation the co-chaperone CHIP emerged as a central player because it acts as a chaperone-associated ubiquitin ligase [5]. CHIP binds to the carboxy-termini of Hsc70 and Hsp70 through a tetratricopeptide repeat (TPR) region and uses a U-box for an interaction with ubiquitin conjugating enzymes mainly of the Ubc4/5 family (Figure 1) [6]. By recruiting Ubc enzymes to the chaperone complex CHIP stimulates the ubiquitylation of a broad range of Hsc/Hsp70 clients including signaling proteins such as the glucocorticoid hormone receptor and aggregation-prone pathogenic proteins. Among the latter are for example mutant forms of the CFTR ion channel that cause cystic fibrosis [7], [8] and hyperphosphorylated tau that forms intracellular tangles in Alzheimer patients [9], [10]. In most cases, CHIP-mediated ubiquitylation initiates sorting to the proteasome for degradation. However, CHIP also participates in the lysosomal degradation of plasma membrane proteins [7] and in chaperone-assisted selective autophagy (CASA) which was recently shown to be essential for muscle maintenance [11]. During CASA clients such as the actin anchoring protein filamin are recognized by the autophagic ubiquitin adaptor p62 after CHIP-mediated ubiquitylation. The adaptor, previously linked to the autophagic degradation of ubiquitin-positive protein aggregates [12], [13], [14], triggers the autophagic engulfment of the ubiquitylated client for sorting towards lysosomal degradation [11]. Whether a proteasomal or autophagic degradation pathway is initiated by CHIP is significantly influenced by additional co-chaperones that bind to the chaperone-CHIP complex (Figure 1). The co-chaperone BAG-1, for example, facilitates proteasomal degradation, because it interacts with the proteasome through a ubiquitin-like (UBL) domain and thus stimulates the docking of the chaperone-CHIP complex at the proteasome [15], [16]. BAG-3, on the other hand, recruits p62 to the chaperone-CHIP complex, which leads to client degradation via the autophagosome-lysosome pathway (Figure 1) [11]. Intriguingly, both BAG-domain co-chaperones bind to the amino-terminal ATPase domain of Hsc/Hsp70 in a mutually exclusive manner. Competitive binding of BAG-1 and BAG-3 to the chaperone-CHIP complex thus seems to represent a molecular switch between chaperone-assisted proteasomal and autophagic degradation [17].

Besides BAG-1 and BAG-3 also the co-chaperone HspBP1 is able to bind to the amino-terminal ATPase domain of Hsc/Hsp70 when CHIP occupies the carboxy-terminus (Figure 1). HspBP1 binding to the chaperone complex interferes with the ubiquitin ligase activity of CHIP and thereby abrogates chaperone-assisted degradation [18]. Indeed, HspBP1 was shown to be essential for CFTR maturation because it prevents the premature degradation of the ion channel at the endoplasmic reticulum [18]. The findings illustrate the importance of the co-chaperone network for balancing protein folding and protein degradation activities of Hsc70 and Hsp70 during proteostasis.

Despite our increasing understanding of the biochemical and cell biological functions of different co-chaperones, we know very little about how coordinated alterations of multiple components of the co-chaperone network could lead to dynamic adaptations of the proteostasis machinery. Here we gain insights into this question by identifying Hsc/Hsp70 co-chaperones that are essential for antigen processing in immune cells and by elucidating changes in the expression and localization of these co-chaperones during immune cell maturation.

When an immune response is triggered, dendritic cells (DCs) and macrophages mature into fully competent antigen-presenting cells able to activate T cells [19]. Endogenous and viral proteins are converted to antigenic peptides by the ubiquitin-proteasome system (UPS). The peptides are loaded onto MHC class I complexes and presented on the cell surface for T cell activation [20]. Newly synthesized defective ribosomal products (DRiPs) are considered as the main source of these antigenic peptides [21], [22]. DRiPs are rapidly degraded forms of otherwise metabolically stable proteins that are defective because of errors in transcription, translation, or folding [23], [24]. Although it was initially postulated that up to 30% of newly synthesized proteins fall into this category and DRiPs could thus broadly represent the translated proteome [23], the extent of DRiP formation remains controversial [25], [26]. In any case, ubiquitylated DRiPs transiently accumulate as protein aggregates during the maturation of mouse bone marrow derived dendritic cells (BMDCs) and human Langerhans cells [21], [27], [28], [29]. These aggregates resemble aggresomes, which form in diverse cell types due to an active sequestration of ubiquitylated polypeptides when the degradation capacity is exceeded, e.g. upon proteasome inhibition or overexpression of aggregation-prone proteins [30]. Accordingly, the term ‘dendritic cell aggresome-like induced structures’ (DALIS) was coined for the aggregates [29]. DALIS formation peaks between 8–12 h after the induction of maturation and depends on ongoing protein synthesis, consistent with an incorporation of DRiPs [21], [29]. Moreover, DALIS formation coincides with a delayed presentation of antigens on the cell surface, which led to the proposal that DALIS represent functional sites in DCs for the storage and regulated processing of antigens [21], [31], [32]. This could allow DCs to coordinate maturation and antigen presentation during their migration to the lymph nodes, based on an active sequestration of ubiquitylated polypeptides throughout an initial ‘aggregation’ phase and the ability to dissolve aggregates in the following ‘presentation’ phase. Yet, underlying mechanisms remained largely elusive. Noteworthy, Hsc/Hsp70 and CHIP have been detected in DALIS, pointing to an intimate involvement of the chaperone machinery in antigen processing at these sites [21], [29]. However, the relevance of the observed co-localization with regard to DALIS formation and disassembly has been unclear so far.

Here we demonstrate that the co-chaperones CHIP and BAG-1 are essential for DALIS formation in mouse DCs and macrophages. Moreover, the ubiquitin ligase activity of CHIP in these cells is under control of the CHIP-inhibitor HspBP1. The expression of the inhibitor increases during the early ‘aggregation’ phase, followed by a decline of expression at late stages when antigens are further processed and presented on the cell surface. The late ‘presentation’ phase is also characterized by an induction of stress-inducible Hsp70 and of the degradation-stimulating co-chaperones BAG-1 and BAG-3. These alterations apparently facilitate the clearance of aggregates at late time points after immune stimulation. Defined changes of the Hsc/Hsp70 chaperone/co-chaperone machinery during maturation thus seem to provide a molecular basis for the transient formation of DALIS in professional antigen presenting cells. The observed findings illustrate how cells can influence protein aggregation through dynamic adaptations of the co-chaperone network. Such adaptations may also underlie proteostasis in other cell types, i.e. postmitotic muscle and neuronal cells that are vulnerable to the cytotoxic consequences of protein aggregation.

## Results

### DALIS represent detergent-insoluble aggregates of ubiquitylated proteins

**Figure 2:**
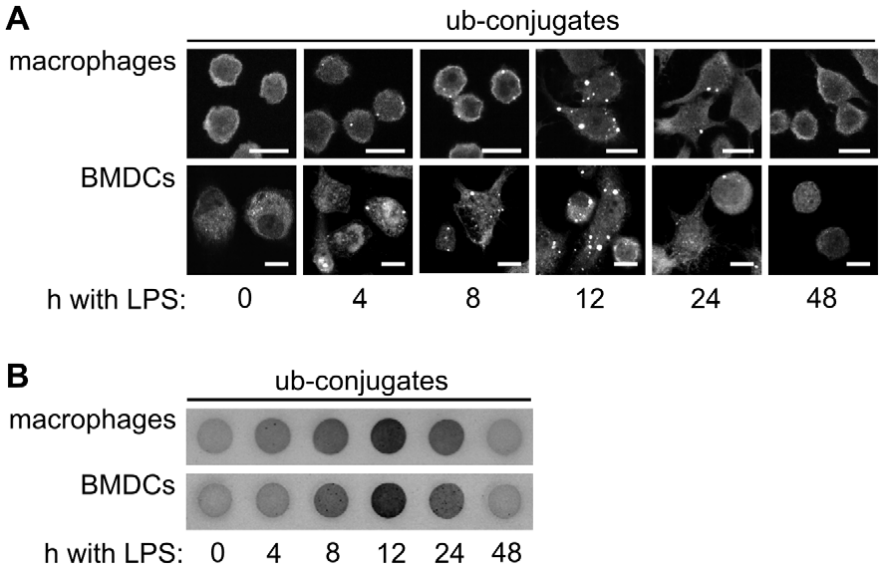
DALIS are detectable by immune fluorescence and a filter trap assay. (A) Following addition of LPS to RAW309 macrophages and mouse BMDCs ubiquitin conjugates were detected by immune fluorescence using the FK2 antibody. Transient DALIS formation peaks around 12 h after LPS addition. Scale bars: 10 µm. (B) Immune cells were lysed at the indicated time points during LPS-induced maturation in detergent containing buffer and passed through a nitrocellulose filter. Detergent insoluble ubiquitin conjugates were detected on the filter with the FK2 antibody. 50 µg of cell lysate were passed through each dot.

Addition of bacterial lipopolysaccharides (LPS) to the mouse macrophage cell line RAW309 and to mouse BMDCs led to the transient accumulation of DALIS, which were detectable by immune fluorescence using the FK2 antibody that specifically recognizes ubiquitin conjugates (Figure 2A). DALIS accumulated during an early aggregation phase between 0 and 12 h, followed by a presentation phase between 12 and 48 h during which DALIS were completely removed.

As an additional method for the detection and quantification of DALIS we adopted a filter trap assay, previously used to verify pathogenic protein aggregation in neuronal cells [33]. Following lysis of immune cells in the presence of high amounts of detergent, lysates were passed through a nitrocellulose filter, leading to the retention of detergent insoluble material. Retained ubiquitin conjugates were finally visualized by probing the filter membrane with the FK2 antibody (Figure 2B). The biochemical assay confirmed the transient accumulation of ubiquitin conjugates as detergent insoluble aggregates in mouse macrophages and BMDCs with a peak at around 12 h after LPS addition.

### Diverse co-chaperones and components of the proteostasis machinery localize to DALIS

**Figure 3:**
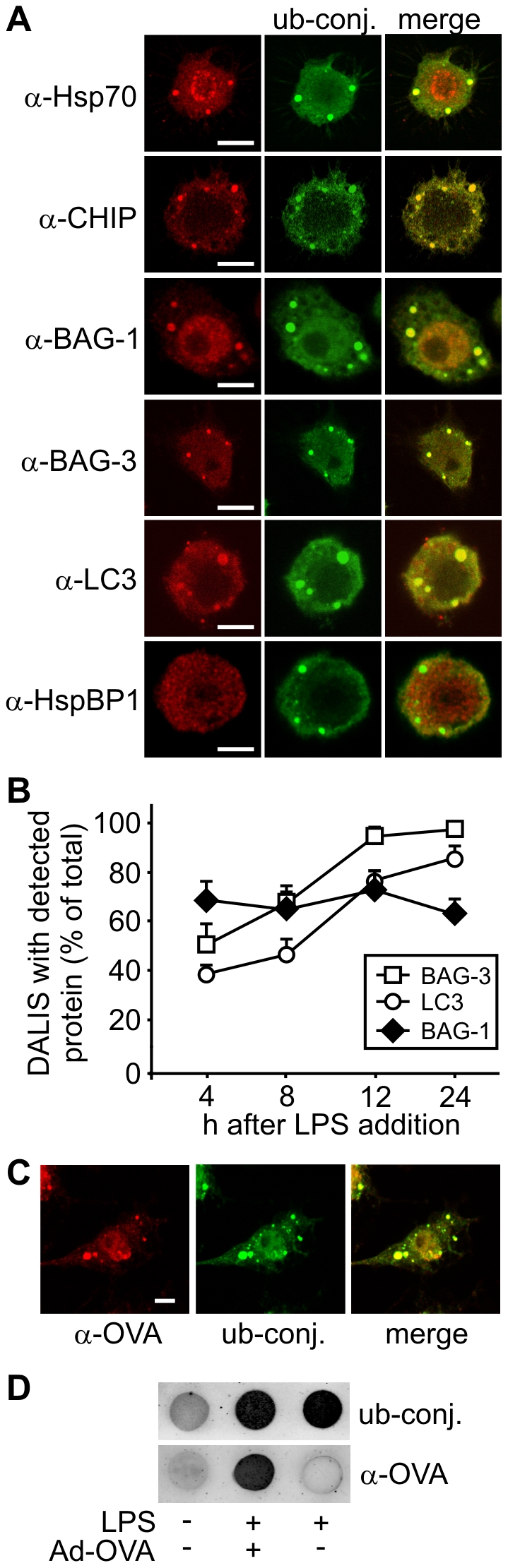
Diverse proteostasis components co-localize with antigens in DALIS. (A) Using specific antibodies against diverse proteostasis components Hsp70, CHIP, BAG-1, BAG-3, and LC3 were detected in DALIS between 12 and 24 h after LPS addition to RAW309 macrophages. In contrast, the co-chaperone HspBP1 did not colocalize with ubiquitin conjugates in DALIS. DALIS were detected with the FK2 antibody that recognizes ubiquitin conjugates (‘ub-conj.’). Scale bars correspond to 5 µm. (B) The amount of DALIS that stained positive for BAG-1, BAG-3 and LC3 were quantified at the indicated time points after LPS addition. Error bars represent sem from three independent experiments. (C) RAW309 macrophages were infected with an ovalbumin expressing adenovirus and stimulated with LPS for 12 h prior to immune fluorescence. Ovalbumin was detected in DALIS with a specific antibody (‘α-OVA’), while ubiquitin conjugates (‘ub-conj.’) were visualized with the FK2 antibody. Scale bar: 5 µm. (D) RAW309 macrophages were infected with Ad-OVA and stimulated with LPS for 12 h. Control cells were left untreated or treated with LPS only. Cells were lysed in detergent containing buffer and 50 µg of total protein were passed over a nitrocellulose membrane. DALIS were detected with FK2 antibody (‘ub-conj.’) and ovalbumin with a specific antibody (‘α-OVA’).

In order to gain insight into the molecular mechanisms underlying the transient formation of DALIS we sought to identify components of the proteostasis machinery, which co-localize with these aggregates. Consistent with previous observations stress inducible Hsp70 and the chaperone associated ubiquitin ligase CHIP were detectable in DALIS (Figure 3A) [21], [29]. Moreover, a central role of the Hsc/Hsp70 chaperone machinery in regulating DALIS formation is further supported by the detection of the co-chaperones BAG-1 and BAG-3 in these aggregates (Figure 3A). Noteworthy, the BAG domain co-chaperones associated with DALIS with different kinetics. While BAG-1, previously shown to facilitate proteasomal degradation [34], was detectable in about 60–70% of DALIS at all time points after LPS-induced maturation, localization of the autophagy-stimulating BAG-3 in DALIS increased from about 50% at 4 h of maturation to about 100% at 12 h (Figure 3B). A constantly increasing association with DALIS was also observed for the autophagosome marker LC3 (Figure 3B). The findings suggest that chaperone-assisted selective autophagy contributes to the clearance of aggregates at late maturation stages.

The CHIP-inhibitor HspBP1 did not localize to DALIS during immune cell maturation (Figure 3A). Apparently, not every co-chaperone that binds to Hsc/Hsp70 in the cytoplasm is forced into the aggregates, rather a subset of co-chaperones with degradation-stimulating activities specifically localizes to DALIS.

To verify that antigens are initially sequestered in DALIS RAW309 macrophages were infected with a recombinant adenovirus expressing ovalbumin (Ad-OVA), which is processed for presentation on MHC class I molecules in this situation. Ovalbumin was readily detectable in DALIS by immune fluorescence and the filter trap assay at twelve hours after infection (Figure 3C and D). Taken together, the obtained data identify DALIS as an antigen processing compartment that contains a distinct set of proteostasis factors.

### Immune cell maturation is accompanied by significant alterations of the Hsc/Hsp70 chaperone machinery

**Figure 4:**
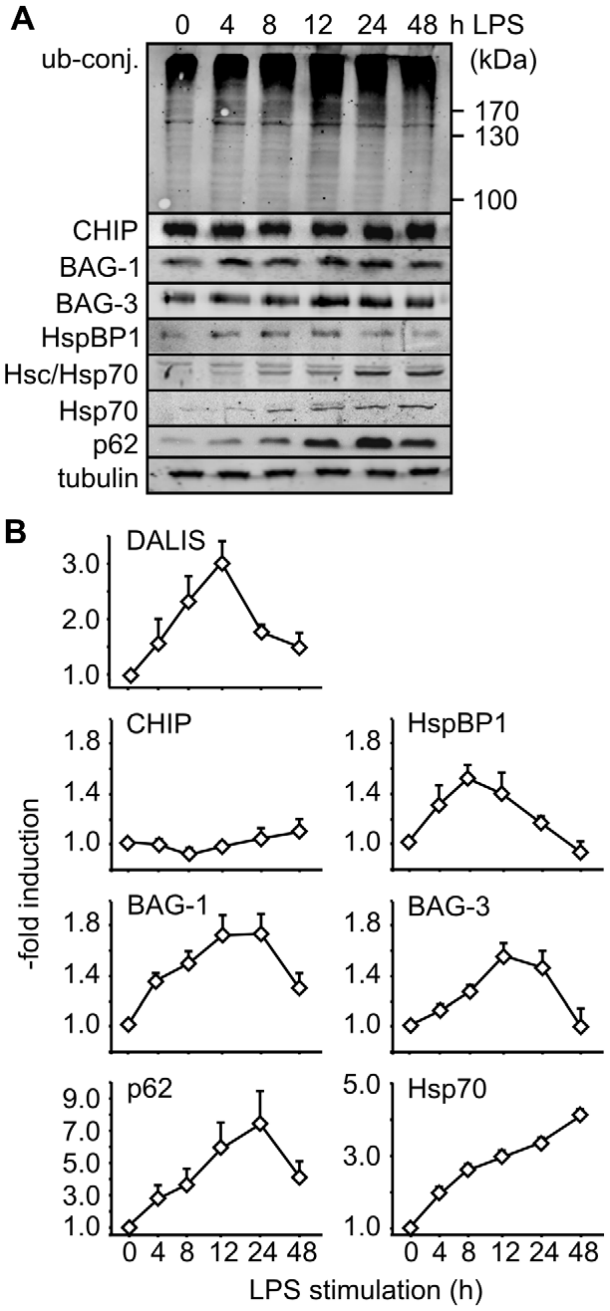
Immune cell maturation involves significant changes of the Hsc/Hsp70 chaperone machinery. (A) Alteration in the levels of ubiquitin conjugates (‘ub-conj.’) and proteostasis components during LPS-induced maturation of RAW309 macrophages were investigated by immune blotting with specific antibodies. (B) Expression of proteostasis components as determined in Figure 4A were quantified and correlated with DALIS formation as determined by filter trap assays (see Figure 2B). Error bars represent sem from at least three independent experiments.

Next we investigated the expression of diverse components of the Hsc/Hsp70 chaperone machinery during immune cell maturation. The cellular concentration of the ubiquitin ligase CHIP was maintained at similar levels during LPS-induced maturation of macrophages and BMDCs (Figure 4A). In contrast, considerable changes of expression were observed for the CHIP-inhibitor HspBP1. Its concentration increased during the first 12 h of maturation, which are characterized by the formation of DALIS, and showed a strong decline at late time points during DALIS removal (Figure 4A and B). Altering the expression level of the CHIP-inhibitor could allow to regulate CHIP-mediated ubiquitylation in the course of immune cell maturation without changing the level of the ubiquitin ligase.

Expression of the degradation-stimulating co-chaperones BAG-1 and BAG-3 and of the autophagic ubiquitin adaptor p62 increased until 24 h after LPS addition, when DALIS level already decreased (Figure 4B). The data suggest that chaperone-assisted proteasomal and autophagic degradation are involved in the clearance of DALIS at late stages of maturation. As BAG-3 and p62 are co-degraded during chaperone-assisted autophagy [11], the observed increase probably represents an underestimate of the induction of these proteins in the course of maturation. Co-degradation would explain the strong decline of both proteins between 24 and 48 h, when DALIS are cleared from the cell.

Also the expression of stress-inducible Hsp70 was strongly upregulated at late maturation stages (Figure 4A and B). This would ensure efficient substrate recognition to initiate chaperone-assisted degradation. Taken together, the observed alterations of the Hsc/Hsp70 chaperone machinery apparently optimize CHIP-mediated processing of antigens during the presentation phase.

### Hsc/Hsp70 co-chaperones control DALIS formation

**Figure 5:**
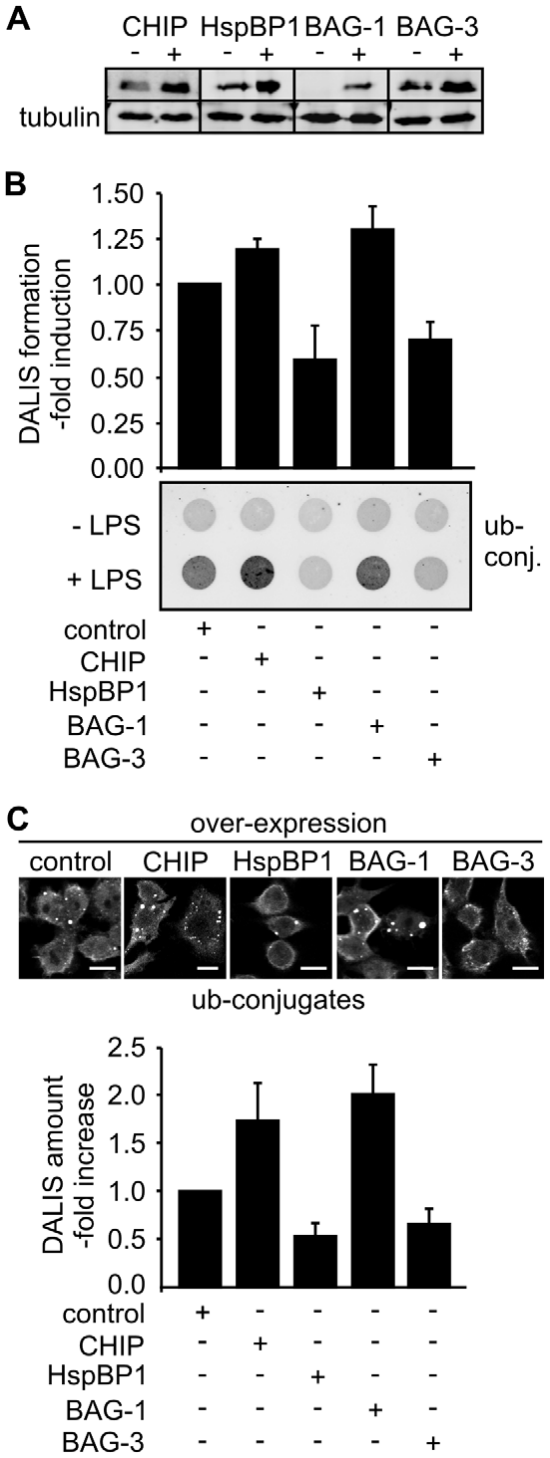
Over-expression of CHIP and BAG-1 increases DALIS formation, whereas HspBP1 and BAG-3 attenuate formation. (A) Co-chaperone levels were determined by immune blotting with specific antibodies following transfection of RAW309 macrophages with equal amounts of empty pcDNA3.1 plasmid (‘−’) or the same vector containing the coding region for the indicated co-chaperone (‘+’). Cell lysates were prepared 24 h after transfection. (B) RAW309 macrophages were transfected with empty plasmid (‘control’) or an equal amount of a co-chaperone encoding plasmid for 24 h followed by LPS addition for 12 h. DALIS formation was analyzed using the filter trap assay. Detergent insoluble ubiquitin conjugates (‘ub-conj.’) were detected on the filter with FK2 antibody and signal intensity was quantified. Value for LPS-stimulated control cells was set to 1. Error bars represent sem from at least three independent experiments. (C) Representative immune fluorescence micrographs of transfected macrophages after over-expression of the indicated co-chaperones for 24 h followed by LPS stimulation for 12 h. Lower panel shows a quantification of obtained data. Number of DALIS observed in control cells were set to 1. Error bars represent sem from at least three independent experiments. Scale bars: 10 µm.

To elucidate how different co-chaperones affect DALIS formation we initially performed transient over-expression of CHIP, HspBP1, BAG-1 and BAG-3, respectively, in RAW309 macrophages (Figure 5A). In all cases, DALIS formation was significantly affected by elevated co-chaperone expression in agreement with a key function of the Hsc/Hsp70 chaperone system in this process (Figure 5B and C). Importantly, co-chaperones could be divided into DALIS-stimulating and -abrogating factors, respectively. While the ubiquitin ligase CHIP and the UBL domain protein BAG-1 stimulated DALIS formation, a significant reduction was observed following overexpression of the CHIP-inhibitor HspBP1 and the autophagy-inducer BAG-3 (Figure 5B and C). The data point to CHIP-mediated ubiquitylation as an initial step in DALIS formation that is regulated by the co-chaperones BAG-1 and HspBP1 in opposite ways. Moreover, BAG-3-triggered autophagy emerges as a process that contributes to aggregate removal.

### CHIP and BAG-1 are essential for DALIS formation

**Figure 6:**
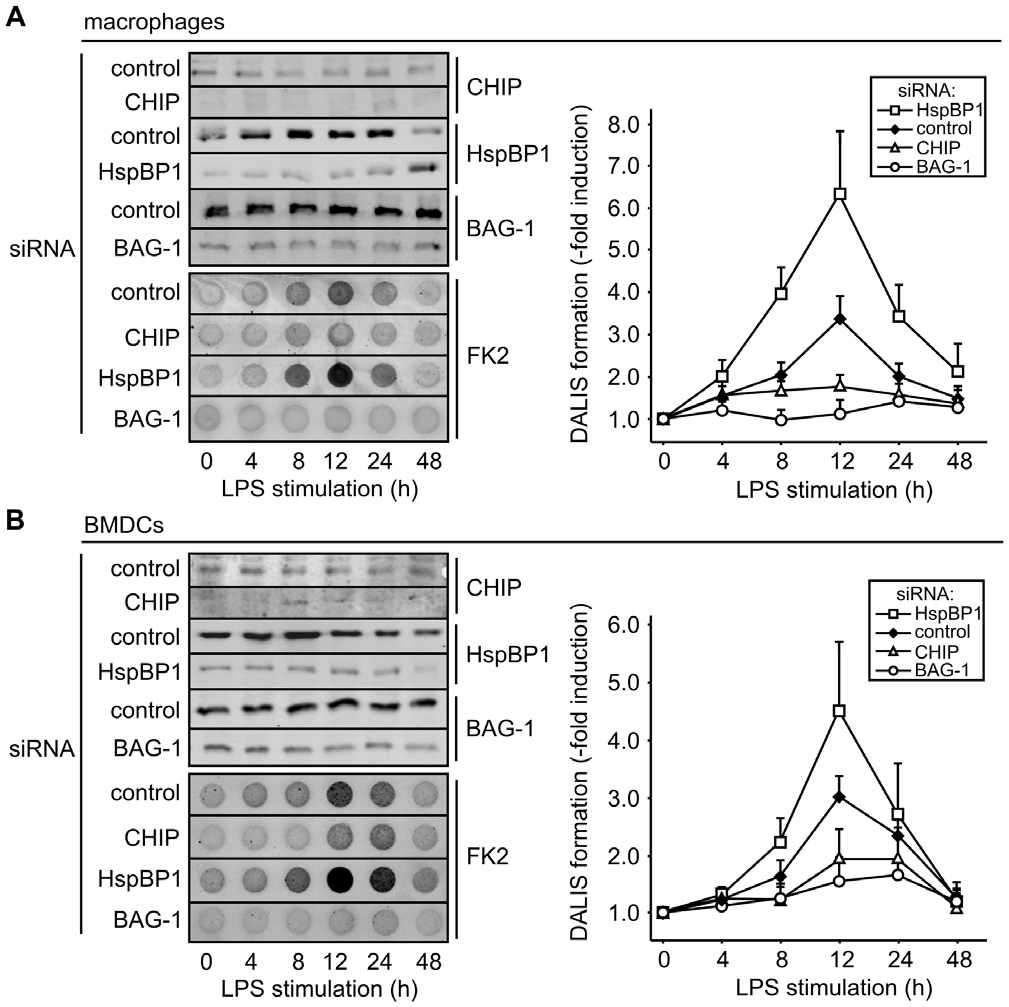
CHIP and BAG-1 are essential for DALIS formation, whereas HspBP1 exerts an attenuating function. (A) Co-chaperones were depleted from RAW309 macrophages by addition of specific siRNAs for 24 h followed by addition of LPS. Control cells received an equal amount of scrambled siRNA. At indicated time points cell extracts were prepared and analyzed by immune blotting or subjected to a filter trap assay for DALIS detection. Signal intensity for detergent insoluble ubiquitin conjugates was quantified. Value at time point zero for control cells was set to 1. Error bars represent sem from three independent experiments. (B) Same as under A, except for the use of mouse BMDCs.

The impact of the Hsc/Hsp70 chaperone machinery on DALIS formation was further investigated in macrophages and BMDCs following siRNA-mediated depletion of individual co-chaperones. Depletion of BAG-3 did not significantly affect the initial accumulation of DALIS nor the clearance of aggregates at late stages in both cell types (data not shown). Although BAG-3 seems to contribute to DALIS removal by facilitating CASA (see data presented above), it is apparently not required for removal. In contrast, depletion of CHIP, BAG-1 and HspBP1 had a strong impact on DALIS formation in macrophages and BMDCs (Figure 6). Reducing the concentration of CHIP in both cell types almost completely abolished protein aggregation during LPS-induced maturation. The data thus identify CHIP as a ubiquitin ligase essential for DALIS formation. Furthermore, depletion of BAG-1 also abrogated DALIS formation (Figure 6). It appears that CHIP-mediated ubiquitylation is essential but not sufficient for client sequestration in DALIS. Subsequent BAG-1 dependent sorting steps are apparently also necessary for sequestration.

**Figure 7:**
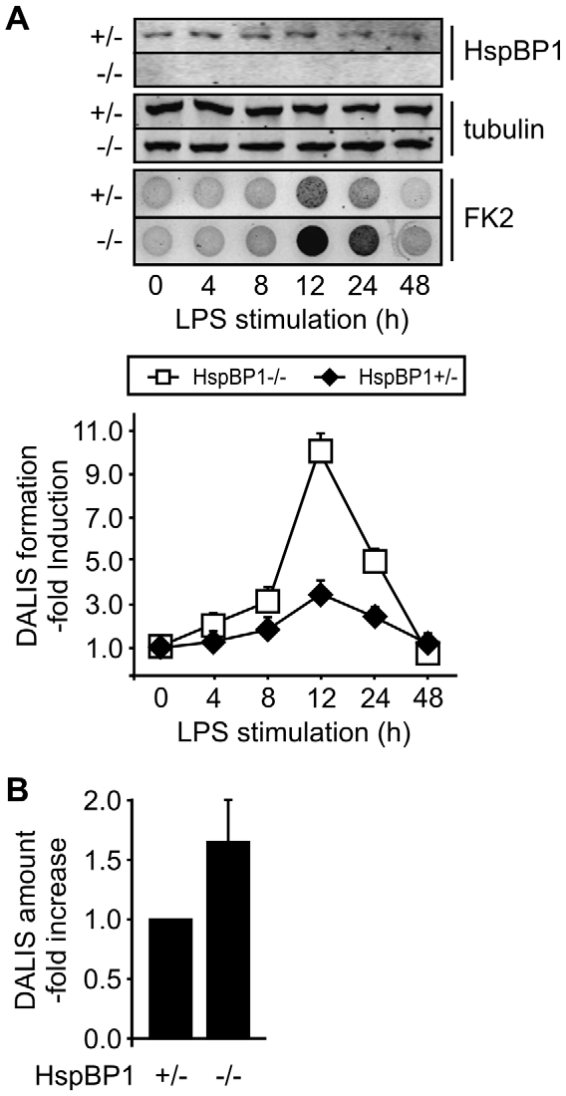
BMDCs isolated from *hspBP1* ^−/−^ mice show increased DALIS formation during maturation. (A) BMDCs were isolated from *hspBP1* ^−/−^ mice and heterozygous siblings and treated with LPS. At indicated time points cell extracts were prepared and analyzed by immune blotting or subjected to the filter trap assay for DALIS detection. Signal intensity for detergent insoluble ubiquitin conjugates was quantified. Value at time point zero for heterozygous cells was set to 1. Error bars represent sem from three independent experiments. (B) DALIS were quantified in BMDCs from *hspBP1*−/− mice and heterozygous siblings by immune fluorescence with FK2 antibody after LPS treatment for 12 h. Error bars represent sem from three independent experiments.

Depleting the CHIP-inhibitor HspBP1 resulted in an increase of DALIS formation in macrophages and BMDCs (Figure 6). Furthermore, similar findings were obtained when BMDCs from *hspBP1* ^−/−^ mice were analyzed. DALIS formation was more than two-fold stimulated in cells lacking the CHIP-inhibitor (Figure 7). The activity of CHIP with regard to DALIS formation is apparently controlled by HspBP1 in professional antigen presenting cells.

### The Hsc/Hsp70 co-chaperone network regulates antigen presentation

**Figure 8:**
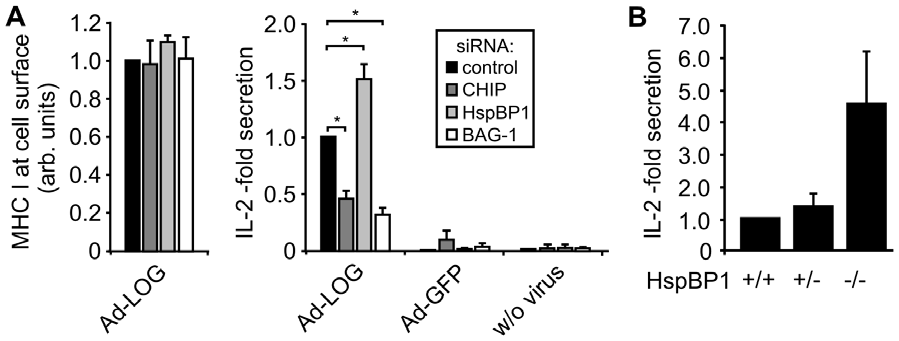
Hsc/Hsp70 co-chaperones regulate MHC class I mediated antigen presentation. (A) After siRNA mediated depletion of the indicated co-chaperones BMDCs were infected with Ad-LOG and stimulated with LPS. After 12 h cell surface localized MHC class I molecules were quantified by FACS analysis (left panel). Fluorescence of control cells, which received a scrambled siRNA, was set to 1. Error bars represent sem from three independent experiments. To monitor antigen presentation cells treated under the same conditions were washed and ovalbumin-specific CD8+ T cells (OT-1) were added. Co-cultivation was performed for 24 h. Presentation on MHC class I complexes was quantified by determining interleukin-2 (IL-2) production of OT-1 cells by ELISA (right panel). Values for control cells receiving a scrambled siRNA were set to 1. Error bars represent sem of at least three independent experiments. Asterisks indicate significance as determined by the two-tailed Student's *t*-test: control/CHIP: *P = 0.018; control/HspBP1: *P = 0.030; control/BAG-1: *P = 0.011 (B) Wild-type, heterozygous *hspBP1* ^+/−^, and homozygous *hspBP1* ^−/−^ mice received an intravenous injection of recombinant influenza A PR/8/43 expressing the SIINFEKL peptide. Spleen cells were isolated after 12 h und incubated with OT-1 cells to monitor antigen presentation on MHC class I molecules based on IL-2 production. Values for wild-type mice were set to 1. Error bars represent sem of three independent experiments.

*In-vitro* cytokine-release assays were performed to elucidate how alterations in the Hsc/Hsp70 co-chaperone network influence antigen presentation. CHIP, BAG-1 and HspBP1, respectively, were depleted in mouse BMDCs using specific siRNAs, followed by infection with a recombinant adenovirus that expresses the immune dominant SIINFEKL peptide of ovalbumin fused to luciferase and GFP (Ad-LOG) [35]. Whereas co-chaperone depletion did not lead to significant changes of the concentration of MHC class I molecules at the cell surface (Figure 8A, left panel), it strongly affected antigen presentation as determined by interleukin-2 (IL-2) production of SIINFEKL specific CD8+ T cells (OT-1), which were co-cultivated with the BMDCs. Depletion of CHIP and BAG-1, shown above to abrogate DALIS formation, impaired antigen presentation (Figure 8A). On the other hand, the depletion of the CHIP-inhibitor HspBP1, which stimulates DALIS formation, led to a ∼1.5-fold increase in presentation efficiency. The components of the Hsc/Hsp70 co-chaperone network that regulate chaperone-assisted degradation apparently control antigen presentation on MHC class I complexes by modulating DALIS formation.

### HspBP1 deficient mice show increased MHC class I dependent immune responses

HspBP1 deficient mice were used to verify the role of the co-chaperone in regulating antigen presentation on MHC class I molecules. Transgenic mice received an intravenous injection of recombinant influenza A virus expressing the SIINFEKL peptide. After 12 h spleen cells were isolated and incubated with OT-1 cells to monitor antigen presentation based on IL-2 production. A ∼4.5-fold increase in IL-2 production was observed for spleen cells isolated from HspBP1 deficient mice (Figure 8B). The *in-vivo* approach confirmed the findings of the *in-vitro* assay and further supported a key regulatory function of the CHIP-inhibitor HspBP1 in the presentation of virally encoded antigens on MHC class I molecules.

## Discussion

**Figure 9:**
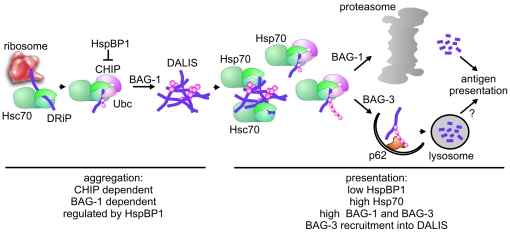
The Hsc/Hsp70 co-chaperone network controls DALIS formation during immune cell maturation. Immune cell maturation can be separated in two distinct phases that are characterized by distinct chaperone environments. During early stages of immune cell maturation, characterized by DALIS formation (aggregation phase), Hsc/Hsp70 binds defective ribosomal products (DRiPs) following their translation. Recruitment of CHIP leads to the ubiquitylation of DRiPs, which provides a sorting signal for sequestration into DALIS. Ubiquitylation activity is regulated by the CHIP-inhibitor HspBP1. DALIS formation also requires BAG-1. Late stages of maturation (presentation phase), which involve antigen presentation and clearance of DALIS, are characterized by decreased HspBP1 levels and induction of Hsp70, BAG-1, and BAG-3. These changes stimulate DRiP processing and presentation. In addition, BAG-3 is recruited to DALIS to facilitate aggregate clearance by chaperone-assisted selective autophagy in cooperation with p62. It remains to be seen whether this autophagy pathway contributes to MHC class I mediated presentation (‘?’).

Molecular chaperones of the Hsp70 family are among the first proteins that associate with translating polypeptides when they emerge from the ribosome exit (Figure 9) [36]. Association is part of a quality control mechanism to detect defective translation products unable to attain their native conformation. In fact, the far majority of these DRiPs are degraded by the ubiquitin/proteasome system in an Hsc/Hsp70 dependent manner [37]. Here we identify the co-chaperone network that regulates Hsc/Hsp70 activity during DRiP processing in professional antigen presenting cells, where DRiPs transiently accumulate in DALIS after LPS-induced maturation. DALIS formation critically depends on the co-chaperone and ubiquitin-ligase CHIP that switches Hsc/Hsp70 activity from protein folding to protein degradation (Figure 9). Previous charaterization of CHIP led to a revision of long held views regarding proteostasis, which invoked a competition between molecular chaperones and degradation systems in order to balance protein folding and degradation [38]. Instead, chaperones apparently fulfill a dual function during proteostasis as facilitators of both, folding and degradation, whereby associating co-chaperones determine the mode of action [5]. The data presented here unravel that also initial steps during the processing of endogenously expressed antigens for presentation on MHC class I molecules rely on a co-chaperone controlled interplay between molecular chaperones and degradation systems. Antigens enter the processing pathway from a chaperone-bound state through ubiquitylation mediated by the chaperone-associated ubiquitin ligase CHIP.

The intimate involvement of the Hsc/Hsp70 chaperone system in initial steps of antigen processing is further supported by the observed impact of the co-chaperones BAG-1 and HspBP1 on DALIS formation. These co-chaperones were previously shown to modulate CHIP activity in opposite ways, with BAG-1 facilitating and HspBP1 abrogating CHIP-mediated degradation [15], [18]. Their antagonistic regulatory function is also evident during immune cell maturation. Ablation or a complete absence of HspBP1 in BMDCs resulted in increased DALIS formation, illustrating that the co-chaperone restricts the activity of CHIP in professional antigen presenting cells. Intriguingly, HspBP1 levels transiently increase during immune cell maturation with a peak that coincides with the peak of DALIS formation (Figure 4). The resultant transient inhibition of CHIP-mediated ubiquitylation may prevent a too excessive diversion of chaperone-bound client proteins onto the aggregation/degradation route when DALIS accumulate. On the other hand, a decline of HspBP1 levels is observed during the late presentation phase. Abrogating CHIP inhibition at this stage would allow ubiquitylation to proceed efficiently and would thus stimulate antigen processing and presentation. The observed changes in HspBP1 expression apparently provide a molecular basis for altering CHIP activity during immune cell maturation with major consequences for antigen processing.

In contrast to HspBP1, the co-chaperone BAG-1 facilitates chaperone-assisted degradation [7], [15], [34]. It acts after CHIP-mediated ubiquitylation by promoting subsequent sorting steps. This activity relies on a ubiquitin-like (UBL) domain that is present at the amino terminus of BAG-1. Through an interaction of this domain with the 19S regulatory particle of the proteasome, BAG-1 was shown to stimulate the docking of Hsc/Hsp70 complexes at the proteasome and to promote CHIP-mediated degradation [16], [34]. Remarkably, however, BAG-1 also facilitates the proteasome-independent degradation of mutant CFTR at the plasma membrane following CHIP-mediated ubiquitylation of the ion channel [7]. The ubiquitin-like domain of BAG-1 may therefore not only be recognized by the 19S particle but also by other ubiquitin-interacting proteins [7]. The finding that CHIP activity is not sufficient for DALIS formation but also requires BAG-1 is in full agreement with an important function of the co-chaperone in post-ubiquitylation sorting steps (Figure 9). The interaction partner that recognizes the UBL domain of BAG-1 in this situation remains to be identified, however. A comparison with another UBL domain protein, namely PLIC-1/ubiquilin, might be revealing here. In this protein the UBL domain provides a binding site for multiple ubiquitin-interacting motif (UIM)-containing proteins, including the S5a subunit of the proteasome, the deubiquitinating enzyme ataxin-3, the co-chaperone HSJ1, and the sorting factors EPS15 and HRS [39], [40], [41]. Intriguingly, a UBL-mediated interaction of PLIC-1 with EPS15 was shown to be required for aggresome formation [39], [41]. Therefore, Heir et al. (2006) proposed a dual function for the UBL domain of PLIC-1 in both proteasomal sorting and transport to aggresomes by competetive UBL-UIM interactions. Whereas PLIC-1 would usually interact with proteasomal S5a, high levels of protein aggregation would clog the proteasome and thus favor UBL-UIM interactions that promote deposition of ubiquitylated clients in aggresomes. A transient inactivation of the proteasome, as recently observed during aggresome formation in fibroblasts [42], may contribute to the observed shift. It is conceivable that a similar dual function underlies the essential role of BAG-1 in DALIS formation during the early phase of immune cell maturation. At late stages proteasome activity would be restored and BAG-1 could then facilitate proteasome recruitment to the aggregates for efficient disassembly (Figure 9).

The observed induction of BAG-3 and p62 and the recruitment of BAG-3 into DALIS at late maturation stages suggest that chaperone-assisted selcective autophagy contributes to the clearance of DALIS. BAG-3 is able to facilitate the interaction of the autophagic ubiquitin adaptor p62 with chaperone complexes and in this way triggers the engulfment of ubiquitylated clients by a phagophore membrane as an initial step towards lysosomal degradation [11], [17]. BAG-3 that is localized in DALIS may in a similar manner initiate the autophagic disposal of the aggregates. It is noteworthy, however, that BAG-3 depletion did not significantly impair the disposal of DALIS. This might be explained by a compensatory upregulation of proteasomal degradation in this situation, because BAG-3 depletion was shown to induce BAG-1 assisted degradation by the proteasome [17]. MHC class I molecules are considered to present primarily proteasomal degradation products. Yet, recent evidence suggests that autophagy stimulates MHC class I presentation [43]. It is thus conceivable that BAG-3 and p62 mediated autophagic clearance of DALIS contributes to antigen presentation.

DALIS were previously proposed to act as antigen storage and processing compartments to coordinate DC migration and antigen presentation [29], [32]. In line with this proposal we observed that siRNA-mediated ablation of CHIP, BAG-1 and HspBP1 had a significant impact on the presentation of endogenously expressed antigens on MHC class I molecules. Consequences for antigen presentation mirrored the effects observed for DALIS formation. While CHIP and BAG-1 ablation impaired both processes, HspBP1 ablation stimulated DALIS formation and increased antigen presentation. *In-vivo* presentation assays using HspBP1 deficient transgenic mice confirmed the regulatory role of the CHIP-inhibitor in antigen presentation. Taken together it becomes apparent that the co-chaperone network, which regulates chaperone-assisted degradation, controls MHC class I mediated antigen presentation through a modulation of DALIS formation.

A transient accumulation of ubiquitin conjugates in protein aggregates has also been observed in non-immune cells following different physiological stimuli and non-phyisological insults. Proteasome inhibition, for example, was shown to result in the formation of a juxtanuclear quality control compartment containing conjugates and proteasomes [44]. Furthermore, non-immune cells respond to oxidative stress with the formation of aggresome-like induced structures, ALIS [28], [42]. Intriguingly, key components of the proteostasis machinery elucidated here, i.e. Hsp70, CHIP, BAG-3 and p62, are induced under oxidative stress und upon proteasome inhibition [11], [45], [46]. Moreover, CHIP deficient mice show an increased sensitivity to oxidative damage [47]. The chaperone-mediated processes that underlie DALIS formation and clearance in immune cells may thus emerge as more general mechanisms for controlling protein aggregation under diverse physiological and pathophysiological conditions.

## Materials and Methods

### Ethics statement

Animal experiments in this study were carried out in accoradance with Tierschutzgesetz §8 Abs. 1 and were approved by "LANUV Nord Rhein Westfalen" (Az: 9.93.2.10.35.07.268). All surgery was performed under anesthesia, and all efforts were made to minimize suffering.

### Antibodies

Commercially available antibodies were used for detection of CHIP (PC711, Calbiochem), HspBP1 (FL-4, Delta Biolabs for immune fluorescence; anti-HspBP1, Transduction Laboratories for immune blotting), ubiquitin conjugates (FK2, Biotrend), BAG-1 (C16, Santa Cruz), BAG-3 (Proteintech Group), p62 (Progen and Dianova) Hsc/Hsp70 (SPA-820, StressGen), Hsp70 (SPA-812, StressGen), ovalbumin (Pierce), LC3 (MBL), and tubulin (Sigma). ELISA was performed with IL-2 antibody (eBioscience). Fc receptors were blocked with 2.4G2 antibody (BD Pharmingen). Cell surface staining for FACS analysis was performed with MHC class I antibody from eBioscience. Secondary antibodies for immune blotting were purchased from LI-COR. Alexa-488 labeled goat anti rabbit and Alexa-546 labeled goat anti mouse secondary antibodies for immune fluorescence were purchased from Invitrogen. Cy2 labeled donkey anti-guinea pig antibody was purchased from Jackson Immuno Research.

### Mice

The generation and characterization of *hspBP1* ^−/−^ mice will be published elsewhere (Christian Rogon and Jörg Höhfeld, manuscript under preparation).

### Cell culture

RAW309 macrophages were grown in DMEM containing 5% heat inactivated fetal calf serum (FCS), 100 IU/ml penicillin, 100 IU/ml streptomycin and 1 mM sodium pyruvate in a 5% saturated CO_2_ atmosphere at 37°C. For preparation of mouse BMDCs bone marrow was rinsed out from femur and tibias of mice in phosphate buffered saline (PBS) with a 0.4 mm needle. It was passed through a 0.4 µm nylon mesh to remove debris and resuspended in IMDM medium containing 10% FCS, 100 IU/ml penicillin, 100 IU/ml streptomycin, and 30% supernatant from the GM-CSF-producing cell line Ag 8653. 5×10^6^ cells were cultured in 3.5 cm diameter Petri dishes. Half of the medium was replaced on day three and six. On day seven most of the cells had acquired typical dendritic morphology, and these cells were used as the source of DCs in subsequent experiments. To induce maturation BMDCs and macrophages were stimulated with 10 ng/ml LPS.

### Isolation of OT-1 cells

For OT-1 cell preparation spleens were removed and minced over a cell strainer. Cells were washed in PBS and resuspended in IMDM. A 10 ml syringe was filled with 0.6 mg nylon wool and was autoclaved. Wool was equilibrated with 2% FCS in PBS, followed by a second incubation with 2% FCS in PBS at 37°C for 45 min. FCS/PBS fluid was eluted and afterwards spleen cell suspension was added. Cells were drained to the bottom of the syringe which was sealed and filled up with medium. Cells were incubated for 1 h at 37°C. After that cells were eluted two times with 20 ml medium. Cells were selected for CD8 positive cells with an autoMACS cell separator (Myltenyi Biotec).

### Transfection

For depletion of co-chaperones in transfection experiments siRNAs were obtained from Qiagen. The following target sequences were selected:

mCHIP: 5′-cacacttgtggcagtgtacta-3′ and 5′-ccggctcttcgtgggccgcaa-3′;

mHspBP1: 5′-cagaaagtccatggcagacaa-3′ and 5′-caggaccgtgaaggcgcacta-3′;

mBAG-1: 5′-cagggagttgactagaagtaa-3′ and 5′-ccgagtcatgttaattggtga-3′.

As control siRNA SiGLO transfection indicator (Dharmacon) was used. 10 ng of siRNA was added in 4 mm electroporation cuvettes to 1×10^6^ RAW309 cells or to 4×10^6^ BMDCs diluted in 100 µl OPTIMEM medium (Invitrogen). Electroporation was performed in an electroporator with additional CE module (Bio-Rad Laboratories). 24 h after electroporation maturation was triggered with 10 ng/ml LPS or by virus infection.

Overexpression in RAW309 macrophages was performed with Fugene HD transfection reagent (Roche Diagnostics) as specified by the manufacturer. 24 h after transfection maturation was stimulated with 10 ng/ml LPS.

### Filter trap assay

For filter trap assays LPS treated cells were lysed in RIPA-buffer (25 mM Tris pH 8, 150 mM NaCl, 0.1% sodium dodecyl sulfate, 0.5% sodium deoxycholat, 1% Nonidet P-40, 10% Glycerol, 2 mM EDTA) and complete protease inhibitor (Roche Diagnostics) by sonication. Protein concentration was determined by Bradford assay and adjusted by comparison of Coomassie-stained SDS-PAGE gels. 50 µg cell lysate was applied to each dot on the nitrocellulose membrane, followed by two washing steps. Membranes were blocked with 2% milk in TBST (25 mM Tris, 137 mM NaCl, pH 7.6, 0.3% Triton X 100) and detergent insoluble ubiquitin conjugates were detected on the membrane with the FK-2 antibody using the Odyssey Infrared Scanning System (LI-COR).

### Immune fluorescence

Cells were washed three times with PBS, followed by fixation in 4% paraformaldehyde for 20 min. After another washing step, cells were blocked in 3% bovine serum albumin for 30 min. Cells were incubated with primary antibodies in a 1∶100 dilution for 1 h. Alexa-labeled secondary antibodies (Invitrogen) were used in a 1∶400 dilution for 1 h. Images were acquired using a Zeiss Axiovert 510 microscope.

### Virus infection and cytokine assays

For detection of ovalbumin in DALIS by immune fluorescence 5×10^4^ RAW309 makrophages were seeded on 12.5 mm cover slips and were infected with 1×10^5^ VP/cell of ovalbumin expressing adenovirus (Ad-OVA) in a volume of 100 µl. 30 min after infection 250 µl of fresh medium was added. After 12 h cells were fixed and stained for immune fluorescence. *In-vitro* cytokine assays were performed with an ovalbumin expressing adenovirus (Ad-LOG) [35]. BMDCs were infected with 1×10^4^ VP/cell in a volume of 500 µl. 30 min after infection 500 µl of fresh medium was added. After 12 h cells were washed three times, diluted in 100 µl and OT-1 cells were added in a ratio of two OT-1 cells to one BMDC. After 24 h supernatant was collected and IL-2 production was analyzed by ELISA. *In-vivo* cytokine assays were performed with a recombinant influenza A virus Pr/8/34 expressing the SIINFEKL peptide. 6–12 month old mice were intravenously infected with 1×10^6^ VP. 12 h after injection spleen cells were isolated and incubated with OT-1-cells for 24 h. IL-2 production was detected by ELISA.

### FACS analysis

At least 1×10^5^ cells per sample were washed with FACS buffer (1% FCS in PBS) and resuspended in 100 µl. Fc receptors were blocked with a 1∶200 dilution of Fc antibody for 20 min. Afterwards cells were incubated for 15 min in a 1∶200 dilution of APC conjugated MHC class I antibody. After another washing step fluorescence was measured with a BD LSRII flow cytometer.
